# Identification and analysis of long non-coding RNAs that are involved in inflammatory process in response to transmissible gastroenteritis virus infection

**DOI:** 10.1186/s12864-019-6156-5

**Published:** 2019-11-04

**Authors:** Xuelian Ma, Xiaomin Zhao, Kaili Wang, Xiaoyi Tang, Jianxiong Guo, Mi Mi, Yanping Qi, Lingling Chang, Yong Huang, Dewen Tong

**Affiliations:** College of Veterinary Medicine, Northwest A&F University, Yangling, Shaanxi 712100 People’s Republic of China

**Keywords:** TGEV, lncRNAs, miRNAs, lncRNA binding proteins

## Abstract

**Background:**

Transmissible gastroenteritis virus (TGEV) infection can cause acute inflammation. Long noncoding RNAs (lncRNAs) play important roles in a number of biological process including inflammation response. However, whether lncRNAs participate in TGEV-induced inflammation in porcine intestinal epithelial cells (IPECs) is largely unknown.

**Results:**

In this study, the next-generation sequencing (NGS) technology was used to analyze the profiles of lncRNAs in Mock and TGEV-infected porcine intestinal epithelial cell-jejunum 2 (IPEC-J2) cell line. A total of 106 lncRNAs were differentially expressed. Many differentially expressed lncRNAs act as elements to competitively attach microRNAs (miRNAs) which target to messenger RNA (mRNAs) to mediate expression of genes that related to toll-like receptors (TLRs), NOD-like receptors (NLRs), tumor necrosis factor (TNF), and RIG-I-like receptors (RLRs) pathways. Functional analysis of the binding proteins and the up/down-stream genes of the differentially expressed lncRNAs revealed that lncRNAs were principally related to inflammatory response. Meanwhile, we found that the differentially expressed lncRNA TCONS_00058367 might lead to a reduction of phosphorylation of transcription factor p65 (p-p65) in TGEV-infected IPEC-J2 cells by negatively regulating its antisense gene promyelocytic leukemia (PML).

**Conclusions:**

The data showed that differentially expressed lncRNAs might be involved in inflammatory response induced by TGEV through acting as miRNA sponges, regulating their up/down-stream genes, or directly binding proteins.

## Background

Virus can activate the inflammatory response by multiple means, including Nuclear factor-kappa B (NF-κB), Jak-STAT, TLRs, T cell receptors (TCRs), NLRs, TNF, RLRs signaling pathway [1–7]. Previous studies have described that TGEV can impair IPECs and trigger inflammatory response [8]. IPECs are the targets for TGEV, and play an important role in the nutrition absorption and inflammatory response against pathogens. The pathogenesis of TGEV is strongly associated with the powerful induction of inflammatory response in host cells. A new study confirmed that the RLRs, TLRs and NF-κB signaling pathways are involved in TGEV-induced inflammatory responses [9].

Non-coding RNAs (ncRNAs), including miRNAs, circular RNAs (circRNAs), as well as lncRNAs, typically do not encode proteins and functionally regulate many biological process [10]. It has been demonstrated that many ncRNAs are involved in inflammatory response in cells [2, 3, 11–15]. In previous study, we determined that the profiles of mRNAs, miRNAs and circRNAs were significantly changed in the IPEC-J2 after TGEV infection. The potential functions of differentially expressed mRNAs, miRNAs and circRNAs were anlyzed and were closely related to inflammatory response [16]. Recently, increasing studies have indicated that lncRNAs play important roles in inflammatory response [17–20]. Therefore, we proposed that lncRNAs also might participate in regulating inflammatory response during TGEV infection.

The lncRNAs play roles in regulating transcription, translation, and protein translocation [21–25]. LncRNAs can regulate translation by interacting with miRNA or act as precursors of miRNA [26–28]. For example, lncRNA SBF2-AS1 acts as a competing endogenous RNA (ceRNA) to modulate cell proliferation via binding with miR-188-5p in acute myeloid leukemia [27]. LncRNA HOTAIR functions as a ceRNA to upregulate Sirtuin 1 (SIRT1) by sponging miR-34a in diabetic cardiomyopathy [29]. LncRNAs can serve as scaffold to bind to different types of proteins or transcription factors at specific domains to activate or inhibit gene transcription. LncRNA H19 decreases the transcriptional activity of p53 [30]. LncRNA SNHG10 facilitates hepatocarcinogenesis and metastasis by modulating its homolog Small Cajal body-specific RNA 13 (SCARNA13) [31]. LncRNAs can also achieve the regulation of the expression of the target genes by recruiting some RNA-binding proteins [32].

This is the first study to demonstrate the expression profiles and regulatory mechanisms of lncRNAs during TGEV infection by NGS methods. The data showed that differentially expressed lncRNAs might be involved in inflammatory response induced by TGEV through acting as miRNA sponges, regulating their up/down-stream genes, or directly binding proteins. This information will enable further research on the TGEV infection and facilitate the development of novel TGE therapeutics targeting lncRNAs.

## Results

### Overview of the Solexa high-throughput sequencing data

**Fig. 1:**
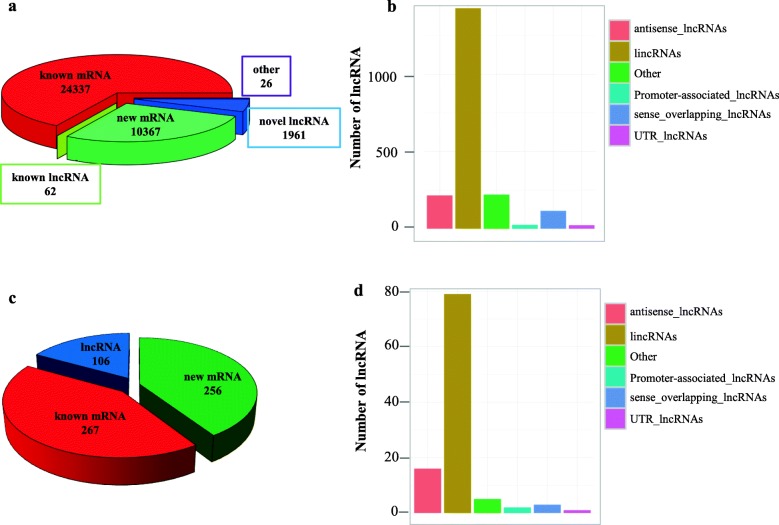
Classification of the assembled transcripts of IPEC-J2 according to their Ensembl code class (pie graphs) detailing lncRNA distribution (bar graphs) of: (**a**) and (**b**) all expressed transcripts; (**c**) and (**d**) transcripts were changed remarkably (fold change > 1.5, and *p* < 0.01)

To investigate the lncRNA expression profiles of TGEV infected IPEC-J2, IPEC-J2 were infected with TGEV strain (TGEV-infected group, indicated by T1 and T2) and the normal IPEC-J2 line (Mock-infected group, indicated by M1 and M2) was used as a control. The RNA-seq was performed with the total RNA extracted from IPEC-J2 infected with 1 MOI TGEV at 24 hpi. Among all mapped transcripts 24,337 (66.22%) were classified as known mRNAs, 10,367 (28.21%) were classified as new mRNAs, 26 (0.07%) were classified as other RNAs (including pseudogenes), and 2023 (5.50%) were classified as lncRNAs (including 62 known lncRNAs and 1961 new lncRNAs) (Fig. 1a and Additional file 1: Table S1). Among them, 215 were antisense lncRNAs, 1427 long intervening/intergenic non-coding RNAs (lincRNAs), 220 other lncRNAs, 24 Promoter-associated lncRNAs, 115 sense overlapping lncRNAs, and 22 UTR lncRNAs (Fig. 1b and Additional file 2: Table S2). The expression levels of 629 transcripts were changed remarkably (fold change > 1.5, and *p* < 0.01). Among all remarkably changed transcripts, 267 (42.45%) were classified as known mRNAs, 256 (40.70%) were classified as new mRNAs, and 106 (16.85%) were classified as lncRNA (Fig. 1c). Among 106 lncRNAs, 16 were antisense lncRNAs, 79 lincRNAs, 5 other lncRNAs, 2 Promoter-associated lncRNAs, 3 sense overlapping lncRNAs, and 1 UTR lncRNAs (Fig. 1d).

### Feature comparison of lncRNA and mRNA

**Fig. 2:**
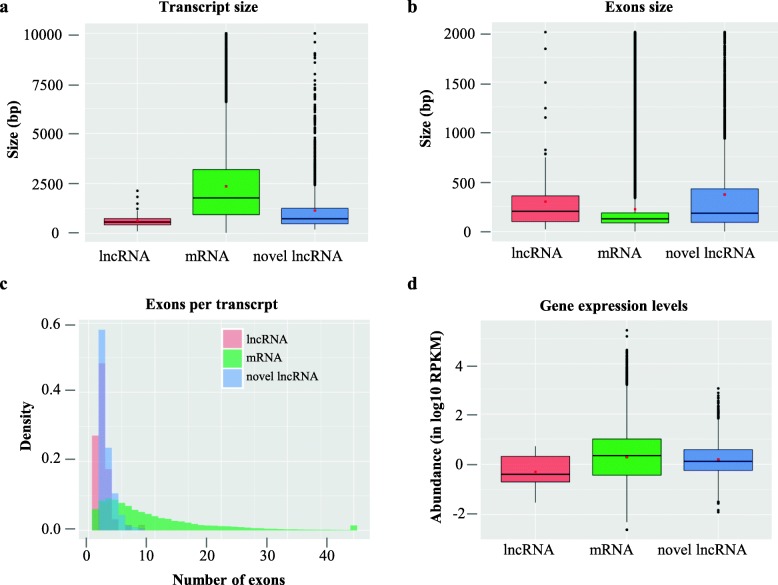
Genomic features of lncRNAs. **a** Transcript sizes of lncRNAs, novel lncRNAs, and mRNAs. **b** Exon sizes of lncRNAs, novel lncRNAs, and mRNAs. **c** Numbers of exons per lncRNAs, novel lncRNAs, and mRNAs. **d** Expression levels (FPKM values) of known lncRNAs, novel lncRNAs, and mRNAs. **a**, **b**, **d** are standard boxplots, which display the distribution of data by presenting the inner fence (the whisker, taken to 1.5× the Inter Quartile range, or IQR, from the quartile), first quartile, median, third quartile and outliers. The means are marked as tan diamonds

In the current study, 2023 lncRNAs and 34,704 mRNAs transcripts were identified. The lncRNAs and mRNAs transcripts were compared for their total length, exon number, exon length, and expression level. We found that known lncRNAs and novel lncRNAs, compared with mRNAs, had significantly shorter transcript length (Fig. 2a), and longer exons (Fig. 2b). These properties were consistent with the lower estimated number of exons for known lncRNAs and novel lncRNAs compared with mRNAs (Fig. 2c). The expression profiles of lncRNAs and mRNAs biotypes were presented as logarithmic distributions. The average mRNA expression level was higher than that of the known lncRNAs and novel lncRNAs (Fig. 2d).

### Profiling of lncRNAs

**Fig. 3:**
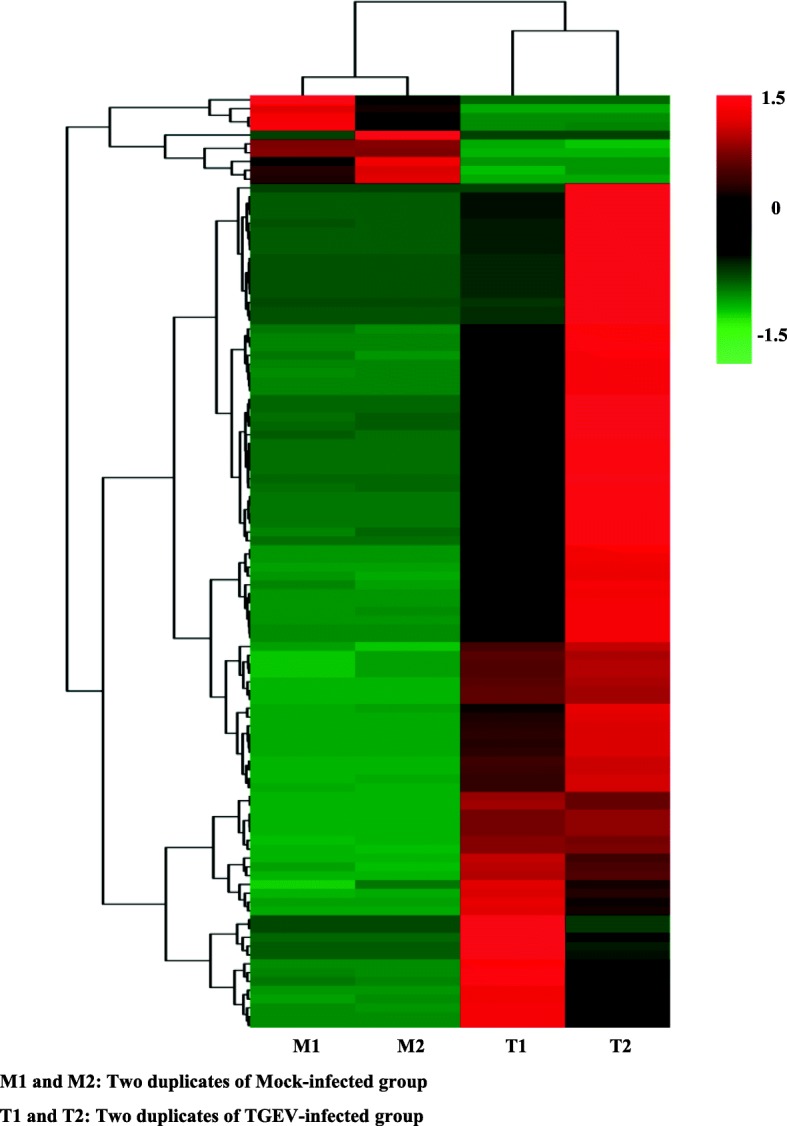
Clustering and Heatmap analysis of differentially expressed lncRNAs (FPKM) across TGEV infection (T1, T2) and Mock infection (M1, M2). Among them 96 lncRNAs were up-regulated and 10 lncRNAs were down-regulated (fold change > 1.5, and p < 0.01)

The differential expression of multiple lncRNAs in TGEV-infected group compared with mock-infected group was observed in Fig. 3. The expression levels of 106 lncRNAs were changed remarkably (fold change ≥2 and ≤ 0.5, FDR < 0.05). Among them 96 lncRNAs were up-regulated and 10 lncRNAs were down-regulated. (Additional file 3: Table S3).

### LncRNAs don’t act as miRNA precursors when TGEV infected

**Fig. 4:**
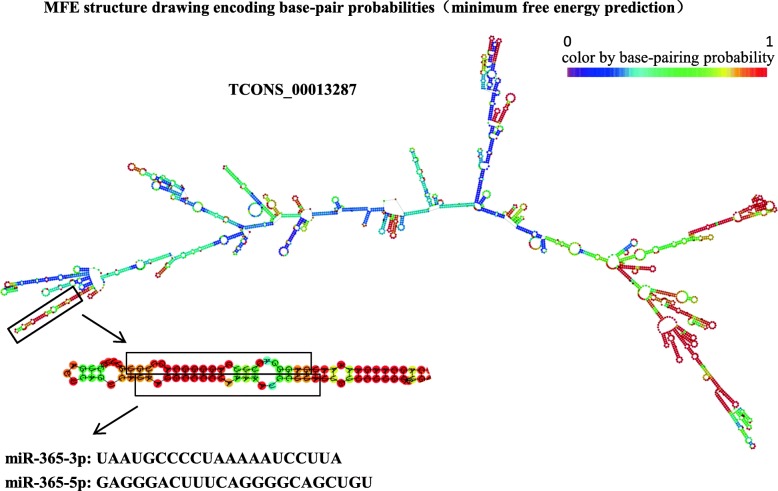
Prediction of miRNA Precursor of lncRNA (take TCONS_00013287 for example)

LncRNAs can be spliced into multiple small RNAs which function as post-transcriptional regulators. To find potential miRNA precursors, lncRNAs were aligned to miRBase (version 21). Our result showed that there were 6 lncRNAs producing precursors of 13 miRNAs possibly (Additional file 4: Table S4). The secondary structures of these lncRNAs and miRNA precursors were predicted via the RNAfold web server (http://rna.tbi.univie.ac.at/cgi-bin/RNAWebSuite/RNAfold.cgi). Figure 4 illustrates the secondary structure of TCONS_00013287, which might release the precursor sequence of miR-365 by an endonuclease cleaving, and form mature miR-365-3p and miR-365-5p finally. The same to their precursors, these 13 miRNAs have no differences between TGEV-infected group and Mock-infected group.

### LncRNAs act as miRNA sponges

**Fig. 5:**
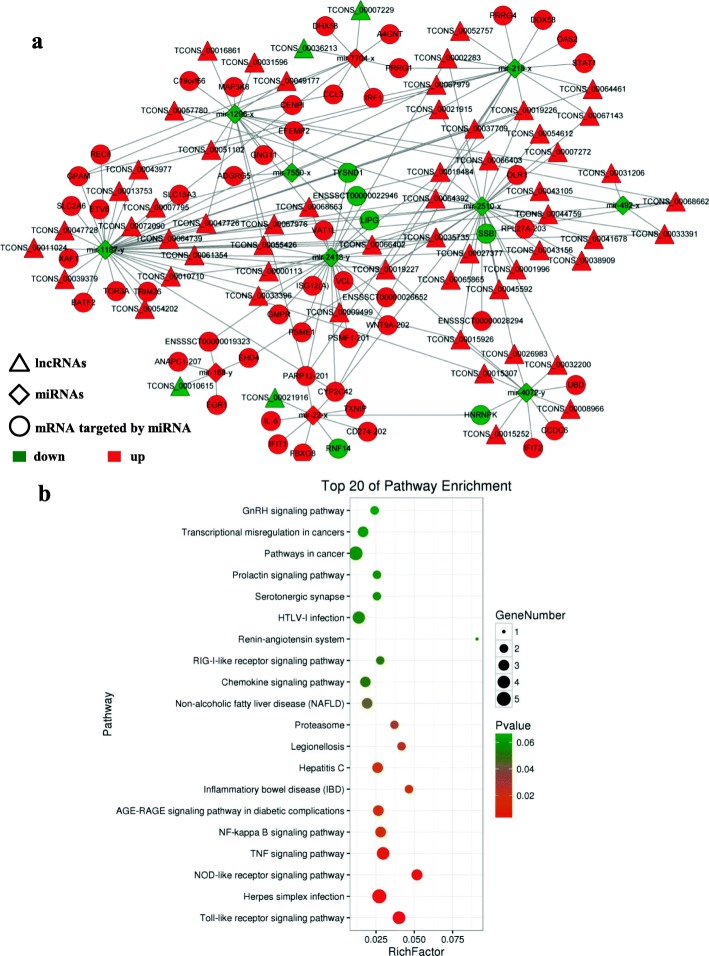
Regulatory network analysis of lncRNA-miRNA-mRNA. **a** The interaction network of lncRNA-miRNA-mRNA. Red and green respectively represent up- and down-regulated genes. Roundness, triangle, and rhombus respectively indicate mRNAs, lncRNAs, and miRNAs. **b** KEGG enrichment analysis of lncRNA-miRNA-mRNA. In this graphic, the degree of KEGG enrichment is assessed by the Rich Factor, *P*-value, and Gene Number. The closer the P-value is to zero, the greater the Rich factor is. The greater the Gene Number is, the more significant the enrichment is

LncRNAs can rescue the translation levels of mRNA via pairing to miRNAs to prevent the binding of miRNAs and mRNA untranslated regions (UTR). In our study, we constructed a lncRNA-miRNA-mRNA expression interaction network combinated with the miRNA sequencing data [16]. A total of 61 differentially expressed lncRNAs and 55 differentially expressed mRNAs targeted 11 differentially expressed miRNAs in the network respectively (Fig. 5a and Additional file 5: Table S5). To find the potential function of these significantly differentially expressed lncRNAs acting as miRNA sponges, kyoto encyclopedia of genes and genomes (KEGG) analysis of the 55 differentially expressed mRNAs was performed and presented. The result showed that these mRNAs were participated in the TLRs signaling pathway, Herpes simplex infection, NLRs signaling pathway, TNF signaling pathway, and NF-κB signaling pathway primarily (Fig. 5b).

### LncRNA-binding proteins

**Fig. 6:**
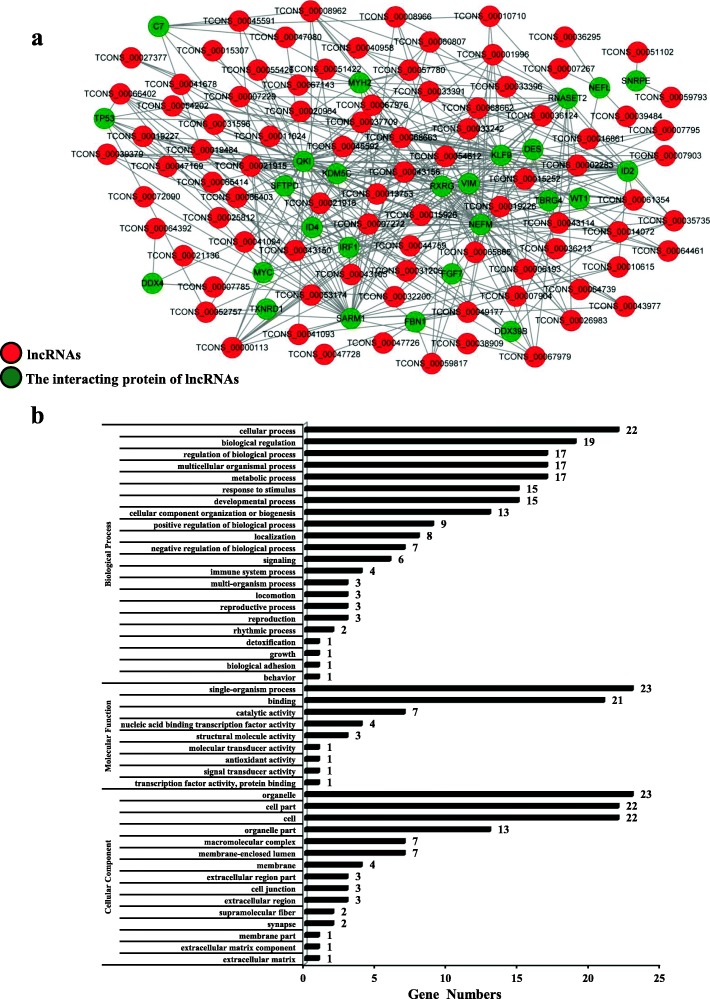
Regulatory network analysis of lncRNA-proteins. **a** The interaction network of lncRNA-proteins. Red and green respectively represent lncRNAs and the interacting proteins of lncRNAs. **b** GO enrichment analysis of the interacting proteins of lncRNAs

We determined lncRNA-protein interactions using the catRAPID omics algorithm [33]. The star rating system of catRAPID helped us rank the results. The score was the sum of three individual values: 1) catRAPID normalized propensity, 2) presence of RNA/DNA binding domains and disordered regions, and 3) presence of known RNA-binding motifs. Three hundred seventy-two lncRNA-protein interactions were predicted for differentially expressed lncRNAs (Fig. 6a and Additional file 6: Table S6); the gene ontology (GO) annotation of 26 proteins with a ranking score > 2 were next explored using GO enrichment analysis. The result showed that 34 lncRNAs interacted with 4 proteins, including complement C7 (C7), inhibitor of DNA binding 2 (ID2), MYC proto-oncogene (MYC), interferon regulatory factor 1 (IRF1), which involve in immune system process (Fig. 6b).

### Up- and down-stream genes of differentially expressed lncRNAs

**Fig. 7:**
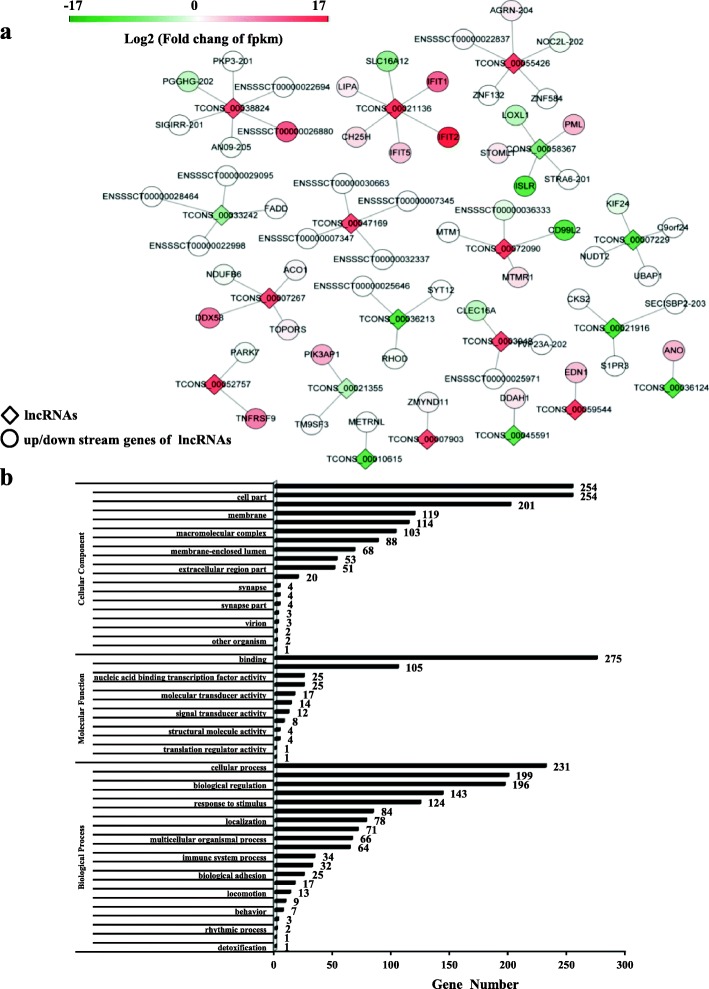
Regulatory network analysis of up- and down-stream genes of lncRNAs. **a** The interaction network of lncRNAs and their up- and down-stream genes. **b** GO enrichment analysis of up- and down-stream genes of lncRNAs

We predicted the up- and down-stream genes of differentially expressed lncRNAs (100 K). Four hundred forty-three genes were obtained, some of which are shown in Fig. 7a and Additional file 7: Table S7. GO analysis was conducted to enrich up- and down-stream targets of differentially expressed lncRNAs (http://www.geneontology.org/). The results exhibited that the 34 up- and down-stream targets of differentially expressed lncRNAs were primarily enriched in immune system process (Fig. 7b).

### Validation of lncRNAs by quantitative real time polymerase chain reaction (q RT-PCR)

**Fig. 8:**
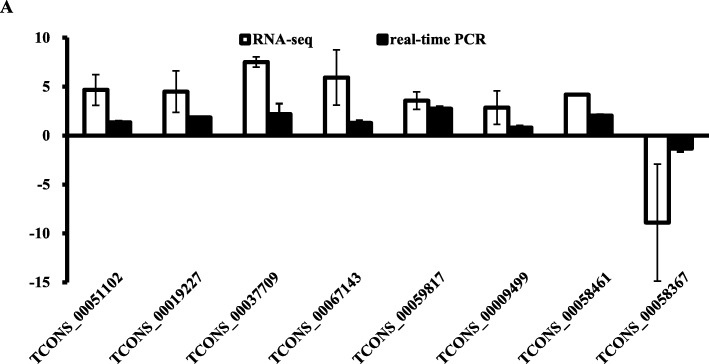
qRT-PCR validation of lncRNAs. The fold change was determined normalized toβ-actin using the 2^-ΔΔCt^ method. The data from qRT-PCR are shown as mean ± standard deviation (S.D.)

To validate the RNA-seq results of differentially expressed lncRNAs, we tested the expression levels of them using qRT-PCR. The fold changes of 8 lncRNAs in TGEV-infected cells were referred to that in mock-infected cells. The results indicated that our sequencing results were accurate. See Fig. 8 and Additional file 3: Table S3.

### Function analysis of the antisense lncRNA TCONS_00058367

**Fig. 9:**
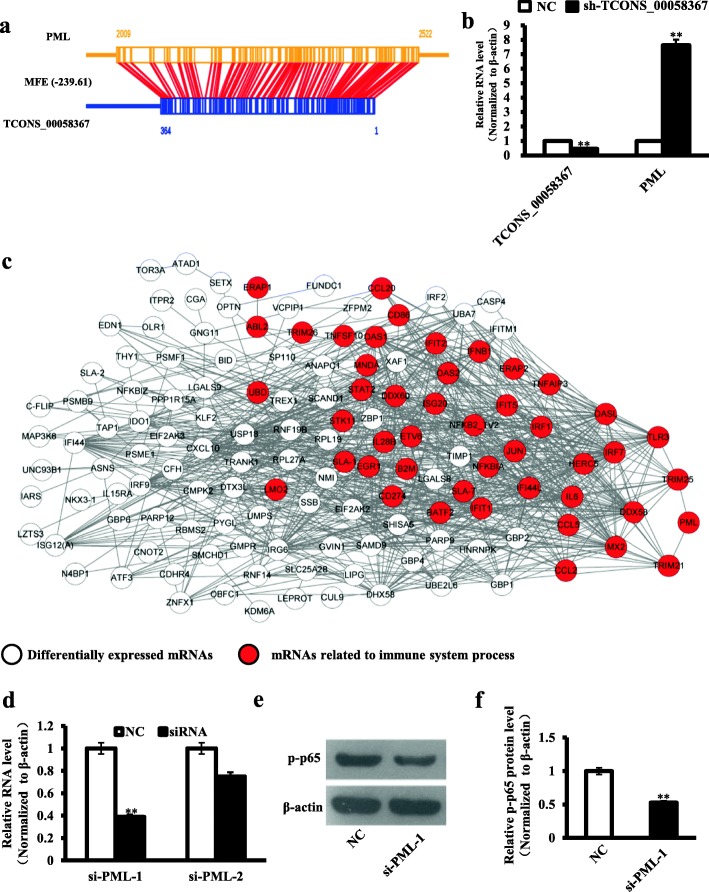
Function analysis of the antisense lncRNA TCONS_00058367. **a** The pattern diagrams showed that lncRNA TCONS_00058367 was located in physical contiguity PML (MFE = − 239.61). **b** Knockdown effect of si-TCONS_00058367 on TCONS_00058367. The relative levels of TCONS_00058367 were measured by qRT-PCR (normalized toβ-actin and in reference to the control). **c** The regulatory relationship between PML and other differentially expressed mRNAs related to immune system process (red). **d** Knockdown effect of si-PML-1 and si-PML-2 on PML. The relative levels of PML were measured by qRT-PCR (normalized toβ-actin and in reference to the control). **e** and (**f**) The effects of si-PML-1 on p-p65. Data represent mean ± S.D. of three independent experiments. **p < 0.01 in comparison with the control

The software RNAplex [3] (http://www.tbi.univie.ac.at/RNA/RNAplex.1.html) was used to predict the complementary correlation of antisense lncRNA and mRNA. The prediction of best base pairing was based on the calculation of minimum free energy (MFE) through thermodynamics structure. The result showed that lncRNA TCONS_00058367 was located in physical contiguity PML (MFE = − 239.61) (Fig. 9a). PML is a nuclear protein that forms sub-nuclear structures termed nuclear bodies associated with transcriptionally active genomic regions. Previous studies have confirmed that PML promotes TNFα-induced transcriptional responses by promoting NF-κB activity. NF-κB signaling pathway plays an important role during TGEV- induced inflammatory response. The antisense lncRNA TCONS_00058367 was down-regulated in TGEV-infected group, and PML was up-regulated in TGEV-infected group. To further understand the regulatory relationship between TCONS_00058367 and PML, IPEC-J2 cells were transfected with shRNA of TCONS_00058367 (sh-TCONS_00058367) (or negative control). The TCONS_00058367 level was down-regulated by sh-TCONS_00058367, while the PML level was up-regulated by sh-TCONS_00058367 (Fig. 9b). The STRING database (version 10.0) was used to further understand the regulatory relationship between PML and other differentially expressed mRNAs related to inflammation process (Fig. 9c and Additional file 8: Table S8). p65 is a subunit of nuclear factor NF-κB. The phosphorylation of p65 is a very significant symbol of NF-κB signaling pathway activity. To explore the function of PML in the process of TGEV induced NF-κB activation, The siRNA of PML (or negative control) were transfected into IPEC-J2 cells respectively, then infected with TGEV at 1 MOI for 24 h. The PML level was down-regulated by si-PML-1 significantly (Fig. 9d). p-p65 was decreased by si-PML-1 (Fig. 9e and f). The siRNA sequences were shown in Additional file 9: Table S9.

## Discussion

LncRNAs have been reported to be involved in the coronavirus infections [20, 34], but the roles of lncRNAs during TGEV induced inflammation response have not yet been elucidated. In our study, NGS techniques were used to investigate the lncRNA expression profiles of TGEV infected IPEC-J2. Among the transcripts of IPEC-J2 obtained in our study, a total of 2023 lncRNAs across the entire genome were screened after sequencing and bioinformatics analysis. These lncRNAs were characterized by shorter transcript length, longer exons, lower estimated number of exons and lower expression levels. These properties were also observed in other reported lncRNAs within the genome [20, 35–37].

In a previous study, TGEV induced inflammatory response via NF-κB signaling pathway, TLRs signaling pathway, NLRs signaling pathway, Jak-STAT signaling pathway, TNF signaling pathway and RLRs signaling pathway [16]. In our study, We identified 106 lncRNAs differential expression between TGEV-infected group and Mock-infected group, reminding us that lncRNAs may be involved in the regulatory process of TGEV infection. LncRNAs can rescue the translation levels of mRNA via pairing to miRNAs to prevent the binding of miRNAs and mRNA UTR. In this study, we found mir-218, which we mentioned earlier, had three target genes, DExD/H-Box helicase 58 (DDX58), Interferon Regulatory Factor 1 (IRF1) and Signal Transducer And Activator Of Transcription 1 (STAT1) that might be involved in inflammatory response. Additionally, ten lncRNAs TCONS_00002283, TCONS_00019226, TCONS_00019227, TCONS_00021915, TCONS_00037709, TCONS_00043977, TCONS_00052757, TCONS_00064461, TCONS_00067143 and TCONS_00067979, which were differentially expressed in TGEV-infected group, were predicted to be targeted by this miRNA, indicating that the lncRNAs may compete with DDX58, IRF1 and STAT1 to affect their expression levels and influence TGEV-induced inflammatory response. Some lncRNAs can directly bind to proteins to regulate the functions of proteins [25, 38]. We determined lncRNA-protein interactions using the catRAPID omics algorithm, the result showed that 34 lncRNAs interacted with 4 proteins, including C7, ID2, MYC, and IRF1, which involve in immune system process. One of the important functions of lncRNA is to act as antisense transcripts of mRNAs or located adjacent to protein coding genes. In our data, many neighbouring genes correspond to compartments of the inflammatory response, such as PML (ENSSSCT00000002141), Interferon Beta 1 (IFNB1) (ENSSSCT00000005691), Radical S-Adenosyl methionine domain containing 2 (RSAD2) (ENSSSCT00000009461), and interferon induced protein with tetratricopeptide repeats 5 (IFIT5) (ENSSSCT00000011440). Previous studies have shown that NF-κB signaling pathway, one of the most important pathways, plays an important role during TGEV- induced inflammatory response [9, 16, 39, 40]. Therefore, changes in the expression levels of genes, which related in NF-κB signaling pathway, might influence the TGEV-induced inflammatory response. The differentially expressed lncRNAs may affect TGEV-induced inflammatory response by affecting NF-κB signaling pathway. It has been proved that PML promotes TNF-α-induced transcriptional responses by promoting NF-κB activity [41]. We further confirm that silencing PML gene expression rescued the TGEV-induced NF-κB activity. In our study, lncRNA TCONS_00058367 was identified as a potential antisense transcript of PML, which suppress transcription of PML. Our work uncovered that lncRNAs might act as regulatory elements of the host inflammatory response when TGEV-infected. While, further efforts should be paied to confirm the present findings.

## Methods

### Research material

The lncRNA expression profile of IPEC-J2 was compared between the IPEC-J2 infected with TGEV (*n* = 2) and Mock group (n = 2). To identify lncRNAs expressed in TGEV infected IPEC-J2, cDNA libraries were constructed and sequenced on the HiSeq 2500 Illumina platform (Illumina, San Diego, CA, USA).

### Strand-specific library construction and sequencing

IPEC-J2 cells were infected with TGEV at 1 MOI for 24 h (indicated by T1 and T2). Meanwhile, the mock infection (indicated by M1 and M2) was carried out. Total RNA was extracted with Trizol reagent (Invitrogen, Carlsbad, CA, US). After total RNA was extracted, ribosomal RNAs (rRNAs) were removed to retain mRNAs and ncRNAs. Following the purification, the enriched mRNAs and ncRNAs were iron-fragmented at 95 °C. Then, reverse transcriptase and random primers were used to generate the first strand cDNA from the cleaved RNA fragments. The second strand DNA was amplified by PCR, QiaQuick PCR extraction kit was used to purify the cDNA fragments, then these fragments were end repaired, poly(A) added, and ligated to Illumina sequencing adapters. The second-strand cDNA was digested by uracil-N-glycosylase (UNG), the products were size selected by PCR amplified, agarose gel electrophoresis, and sequenced using Illumina HiSeqTM 2500 system (Illumina, USA).

### Alignment with reference genome

Reads containing adapters, low quality reads, and rRNA reads were removed. The remaining reads of each sample were then mapped to *Sus scrofa* reference genome (*Sus scrofa* 10.2) by TopHat2 (version 2.0.3.12), respectively.

### Transcripts reconstruction

Cufflinks (V2.2.1), which preferring to the program reference annotation-based transcripts (RABT), was used to reconstruct the transcripts. The influence of low coverage sequencing was fixed through Cufflinks constructing faux reads based on reference. During the end of assembly, similar fragments were removed from all of the reassembled fragments by aligning with reference genes. Then we used Cuffmerge to merge transcripts from different replicates of a group into a comprehensive set of transcripts, and then the transcripts from multiple groups were merged into a finally comprehensive set of transcripts.

### Identification and annotations for novel transcripts

To identify the novel transcripts, all of the reconstructed transcripts were aligned with reference genome and divided into twelve categories using Cuffcompare (V2.2.1). We used the following parameters to identify reliable novel transcripts: the length of transcript was longer than 200 bp and the exon number was more than 2.

### Classification, characterization, and validation of lncRNAs

Two softwares coding-non-coding index (CNCI) (https://github.com/www-bioinfo-org/CNCI) [42] and coding potential calculator (CPC) (http://cpc.cbi.pku.edu.cn/) [43] were used to assess the protein-coding potential of new transcripts by default parameters. The intersection of both results were chosen as long non-coding RNAs.

### Quantification of lncRNA abundance

LncRNA abundance was quantified by RSEM (V1.2.8) and normalized to fragments per kilobase of transcript per million mapped reads (FPKM). The formula is shown as follow:

$$ \mathrm{FPKM}=\frac{10^6C}{NL/{10}^3} $$

C, the number of fragments that are mapped to transcripts; N, the total number of fragments that are mapped to reference genes; L, the number of base pairs of transcript.

### Significance analysis of lncRNAs

The edgeR package (http://www.r-project.org/) was used to identify differentially expressed lncRNAs. A fold change ≥2 and ≤ 0.5, plus a false discovery rate (FDR) <0.05, were identified as significant differentially expressed lncRNAs.

### miRNA precursor prediction

LncRNAs can be spliced into multiple small RNAs which function as post-transcriptional regulators. To find potential miRNA precursors, lncRNAs were aligned to miRBase (version 21). Those with identity more than 90% were selected.

### LncRNA-miRNA interaction

Based on the sequences of lncRNAs, three softwares RNAhybrid (v2.1.2) + svm_light (v6.01), Miranda (v3.3a) and TargetScan (Version:7.0) were used to the candidate target genes. The interaction networks among lncRNA and miRNA were built and visualized using Cytoscape (v3.5.1) (http://www.cytoscape.org/).

### LncRNA cis-regulation analysis

One of the functions of lncRNAs is cis-regulation of their neighboring genes on the same allele. The up-stream lncRNAs which have intersection of promoter or other cis-elements may regulate gene expression in transcriptional or post-transcriptional level. The downstream or 3’UTR region lncRNAs may have other regulatory functions. LncRNAs, which are classified as located in an “unknown region” in Cuffcompare (V2.2.1) were annotated as up-or downstream of a gene. LncRNAs in up/down stream of a gene were likely to be cis-regulators. The interaction networks among lncRNA and up-or downstream genes were built and visualized using Cytoscape (v3.5.1) (http://www.cytoscape.org/).

### Antisense lncRNA analysis

In order to reveal the interaction between antisense lncRNA and mRNA, the software RNAplex [44] (http://www.tbi.univie.ac.at/RNA/RNAplex.1.html) was used to predict the complementary correlation of antisense lncRNA and mRNA.

### GO and KEGG analysis of differentially expressed lncRNAs

GO database (http://www.geneontology.org/) and KEGG database (http://www.genome.jp/kegg/) were used to annotate the pathways. The calculating formula is the same as the previous study [16].The interaction networks among lncRNAs, miRNAs, mRNAs or proteins were built and visualized using Cytoscape (v3.5.1) (http://www.cytoscape.org/).

### Quantification of lncRNAs, miRNAs, and mRNAs using qRT-PCR

According to the manufacturer^′^s instructions, TRIzol reagent was used to extract the total RNA of IPEC-J2 cells, then reverse transcription was carried out using M-MLV reverse transcriptase (Invitrogen, US). qRT-PCR was performed on iQ5 qRT-PCR System (Bio-Rad, US). The primers are shown in Additional file 10:Table S10.

### Western blot analysis

RIPA lysis buffer containing phenylmethylsulfonyl fluoride (PMSF) was used to treat samples to extract the protein, then using BCA Protein Assay Reagent (Pierce, US) to measure the protein concentration. Proteins were separated on sodium dodecyl sulfate-polyacrylamide gel electrophoresis (SDS-PAGE) and transferred onto polyvinylidene difluoride (PVDF) membranes (Millipore, US) subsequently. Block the PVDF membrane with 5% non-fat milk for 2 h at room temperature and then incubate the PVDF membrane with Phospho-NF-κB p65 (p-p65) Rabbit monoclonal antibody (CST, US) overnight at 4 °C and Horseradish peroxidase (HRP)-conjugated secondary antibody (Pierce, US) at room temperature for 1 h subsequently. In the last step, the membrane was developed with enhanced chemiluminescence (ECL) (Promega, US).

### Statistical analysis

SPSS 16.0 was used for statistical analysis. The data are presented as the means ± SEM. Statistical significance was analyzed by unpaired Student^′^s t-test. *p* < 0.05 was defined as statistical significance.

## Supplementary information

**Additional file 1: Table S1.** Classification of the assembled transcripts of IPEC-J2.

**Additional file 2: Table S2.** Classification of the lncRNAs of IPEC-J2.

**Additional file 3: Table S3.** The detailed information of differentially expressed lncRNAs.

**Additional file 4: **Function analysis of the antisense lncRNA TCONS_00058367**.** Prediction of miRNA precursor of lncRNA.

**Additional file 5: Table S5.** The lncRNA-miRNA-mRNA regulation network.

**Additional file 6: Table S6.** The lncRNA-proteins regulation network.

**Additional file 7: Table S7.** The lncRNA-up and down genes network.

**Additional file 8: Table S8.** The interactions of differential expression mRNAs.

**Additional file 9: Table S9.** The sequences of TCONS_00058367 shRNA and PML siRNA.

**Additional file 10: Table S10.** Primers designed for qRT-PCR.

## Data Availability

The raw data were submitted to the National Center for Biotechnology Information (NCBI) Sequence Read Archive (SRA). (https://trace.ncbi.nlm.nih.gov/Traces/sra/sra.cgi?view=run_browser). The accession numbers of the TGEV-infected group (T1, T2) and the Mock-infected group (M1, M2) are No.SRR6447591 and No.SRR6447590.

## Abbreviations

C7: complement C7
ceRNA: competing endogenous RNA
circRNAs: circular RNAs
CNCI: coding-non-coding index
CPC: coding potential calculator
DDX58: DExD/H-Box helicase 58
DF-12: dulbecco’s modified eagle medium (DMEM)/F-12/HAM
ECL: enhanced chemiluminescence
FDR: false discovery rate
FPKM: fragments per kilobase of transcript per million mapped reads
GO: gene ontology
ID2: inhibitor of DNA binding 2
IFIT5: interferon induced protein with tetratricopeptide repeats 5
IFNB1: interferon beta 1
IPEC-J2: porcine intestinal epithelial cells-jejunum 2
IPECs: porcine intestinal epithelial cells
IRF1: interferon regulatory factor 1
KEGG: kyoto encyclopedia of genes and genomes
lincRNAs: long intervening/intergenic non-coding RNAs
lncRNAs: long non-coding RNAs
MFE: minimum free energy
miRNAs: microRNAs
MOI: multiplicity of infection
mRNAs: messenger RNAs
MYC: MYC proto-oncogene
ncRNAs: non-coding RNAs
NGS: next-generation sequencing
NLRs: NOD-like receptors
PML: promyelocytic leukemia
PMSF: phenylmethylsulfonyl fluoride
PVDF: polyvinylidene difluoride
RABT: reference annotation-based transcripts
RLRs: RIG-I-like receptors
RSAD2: radical S-Adenosyl methionine domain containing 2
SCARNA13: small cajal body-specific RNA 13
SDS-PAGE: sodium dodecyl sulfate-polyacrylamide gel electrophoresis
SIRT1: sirtuin 1
STAT1: signal transducer and activator of transcription 1
TCRs: T cell receptors
TGEV: transmissible gastroenteritis virus
TLRs: toll-like receptors
TNF: tumor necrosis factor
TRIM25: tripartite motif containing 25
UNG: uracil-N-glycosylase
UTR: untranslated regions
